# Various clinical scenarios leading to development of the string sign of the internal thoracic artery after coronary bypass surgery: the role of competitive flow, a case series

**DOI:** 10.1186/1749-8090-7-12

**Published:** 2012-01-30

**Authors:** Rudolf Kolozsvari, Zoltan Galajda, Tamas Ungvari, Gabor Szabo, Ildikó Racz, Tamás Szerafin, István Herzfeld, István Edes, Arpad Peterffy, Zsolt Koszegi

**Affiliations:** 1 Department of Cardiology, University of Debrecen, Hungary- place of research conducted; 2 Karolinska Hospital, Department of Thoracic Radiology, Stockholm, Sweden

## Abstract

**Background:**

The left internal mammary artery (LIMA) is the choice for grafting of the left anterior descending coronary artery (LAD). One possible mechanism of the rare graft failure involve the presence of competitive flow.

**Method:**

105 patients who had undergone coronary bypass grafting between 1998 and 2000 were included in this observational study. The recatheterizations were performed 28 months after the operations. The rate of patency the LIMA grafts was determined, and the cases with graft failure were analyzed.

**Results:**

The LIMA graft was patent in 99 patients (94%). Six patients (6%) exhibited diffuse involution of the graft (string sign). The string sign was always associated with competitive flow as the basis of the LIMA graft involution. In one case quantitative re-evaluation of the preoperative coronary angiography revealed merely less than 50% diameter stenosis on the LAD with a nonligated side-branch of the LIMA. At recatheterization in two patients the pressure wire measurements demonstrated only a non-significant decrease of the fractional flow reserve (0.83 and 0.89), despite the 53% and 57% diameter stenosis in the angiogram. Another patient displayeda significant regression of the LAD lesion between the pre- and postoperative coronary angiography (from 76% to 44%) as the cause of the development of the competitive flow. In one instance, a radial artery graft on the LAD during a redo bypass operation resulted in competitive flow in the radial graft due to the greater diameter than that of the LIMA. In a further patient, competitive flow developed from a short sequential part of the LIMA graft between the nonsignificantly stenosed diagonal branch and the LAD, with involution of the main part of the graft to the diagonal branch.

**Conclusions:**

The most common cause of the development of the string sign of a LIMA graft due to competitive flow is overassessment of the lesion of the LAD. Regression of a previous lesion or some other neighboring graft can also cause the phenomenon.

## Background

Prospective angiography in the Bypass Angioplasty Revascularization Investigation trial indicated a 91.6% 1-year patency (< 50% stenosis) for left internal mammary artery (LIMA) grafts. The atherosclerosis of LIMA grafts is rare, and the long-term patency rate of left LIMA-to-left anterior descending (LAD) coronary artery grafts is approximately 90% [1,2].

The high patency of the LIMA-to-LAD graft also leads to an improved clinical outcome. Extensive observational studies showed that patients who received a LIMA-to-LAD graft had a better survival, and experienced fewer adverse cardiac events as compared to patients who received only saphenous venous grafts (SVGs) [3,4].

However, there are increasing reports of LIMA graft failure due to diffuse narrowing.

Barner described the longitudinal thinning of IMA grafts in 1974, referring to it as "disuse atrophy", because the native coronary arteries to which the IMAs were anastomosed appeared to be patent and to have good flow [5]. Several years later, Geha and Bane reported the "distal thread phenomenon" in 2% of IMA grafts 13 months after coronary artery bypass grafting (CABG) [6], correlating it with grafts applied to mildly stenotic recipient coronary arteries. This type of LIMA graft failure was referred to widely as "string phenomenon".

The most common cause of the distal narrowing phenomenon appears to be grafting of the LIMA to a coronary artery with noncritical stenosis (< 50%), but several other causes for this phenomenon have been emerged, including damage during harvesting and mobilization of the LIMA, spasm, inflammation as part of a post-pericardiotomy syndrome or a steal phenomenon arising from a large undivided proximal branch of the LIMA [7,8].

CABG to moderately stenotic target vessels may lead to competitive flow between the native vessel and the graft. Because of individual variations in the effects of stenotic lesions on the coronary flow during resting and exercise conditions and variations in the ability of the LIMA graft to adapt to the flow requirements, there is some controversy as regards the implication of competitive flow on the patency of LIMA bypass grafts [9-11].

Our present aim was to investigate the frequency of string sign of LIMA grafts in a series of patients 2-4 years after bypass surgery and to analyze the cases with regard to the role of competitive flow.

## Materials and methods

### Patient selection

105 patients (82 male, 23 female, mean age: 61 ± 6 years) operated on between 1998 and 2000 were enrolled in this observational study. All of them had received a LIMA graft to the LAD, individually or sequentially with the diagonal branch; 1-3 further distal SVG anastomoses or radial grafts were applied, with an average of 3.1 ± 1 distal anastomoses. The recatheterizations, performed 28 ± 11 months after the operations, were indicated by the clinical symptoms.

The rate of patency of the LIMA grafts was determined, and the cases with LIMA graft failure were analyzed.

### Qualitative and quantitative coronary angiography (QCA)

The native coronary artery stenosis and graft patency were evaluated independently by expert readers according to the American Heart Association guidelines. The most severe stenosis in the luminal diameter of the coronary artery proximal to the anastomotic site was regarded as stenosis of the target coronary branch.

QCA was performed with Philips Integris software from multiple projections in all patients. Reference diameter, minimal luminal diameter, and stenotic diameter were calculated, using the guiding catheter as a scaling device. The most extreme values were chosen for the study. The measurements were carried out both retrospectively on the preoperative coronary angiography and on the index angiography obtained during the recatheterization.

Competitive flow was analyzed by the method of Nakajima et al.: the target coronary branch and anastomotic site are clearly opacified in the native coronary angiography, but not in the graft angiography. Selective LIMA angiography (performed with dedicated IMA catheter, if required) was an inclusion criterion of the study [12,13].

### Fractional flow reserve (FFR) measurement

After the administration of 5000 U of heparin, a left coronary guiding catheter was advanced in the left main coronary ostium. 100 μg of nitroglycerine was administered intracoronarily, and a 0.014-inch sensor-tipped pressure guidewire (Pressure Wire, Radi Medical Systems, Uppsala, Sweden) was advanced to the tip of the guiding catheter. After equal pressures were confirmed at that location, the wire was advanced into a coronary artery. 100 μg of adenosine was administered intracoronarily to induce maximum coronary hyperemia. FFR was calculated by the ratio *Pd/Pa *at maximal hyperemia, where *Pd *is the mean distal pressure in the coronary artery (recorded by the pressure wire) and *Pa *is the mean aortic pressure (recorded by the guiding catheter).

## Results

Six of the 105 patients (6%) exhibited diffuse involution of the graft (string sign). These cases were analyzed individually.

### Case 1

**Figure 1:**
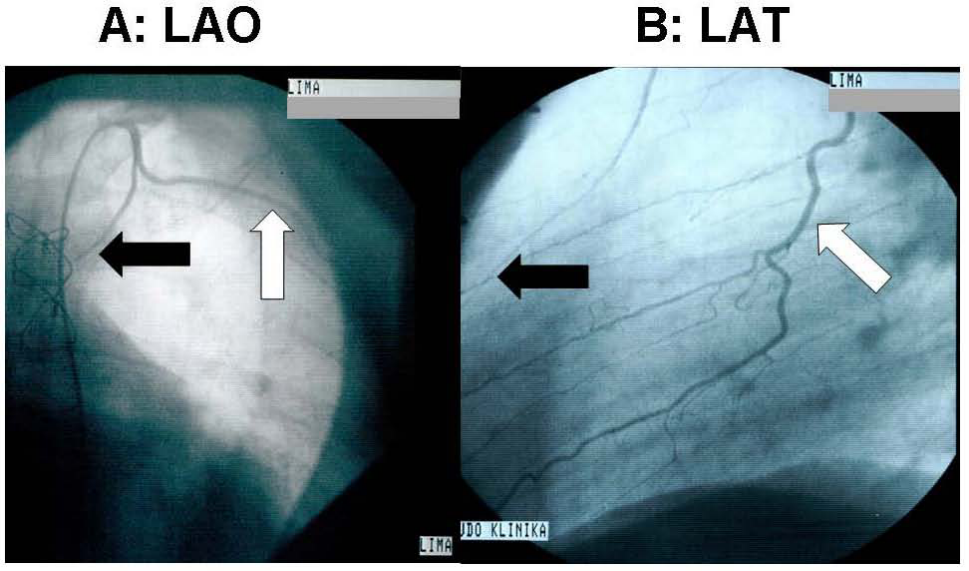
**The side-branch of the mammary graft from left anterior oblique (**A**) and lateral (**B**) views**. (The white arrow indicates the side-branch, and the black arrow shows the string sign of the mammary main branch.).

A 60-year-old male patient who had participated in CABG 4 years previously (LIMA-LAD, SVGA-RCA) underwent repeated coronary angiography because of chest pain and a positive stress test. The angiography revealed an unligated thick side-branch of the left mammary artery with a high run-off, which probably contributed to the string sign of the main branch of the mammary graft (Figure 1). The possibility of an intervention for the occlusion of the side-branch arose, but the significance of the original LAD lesion was questionable, and the patient later became free of angina due to aggressive medical therapy.

### Case 2

**Figure 2:**
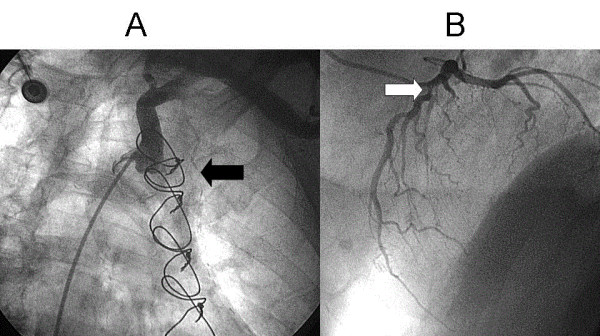
**A: String sign of the left internal mammary artery (black arrow)**. **B**: Lesion of the native left anterior descending artery (white arrow).

**Figure 3:**
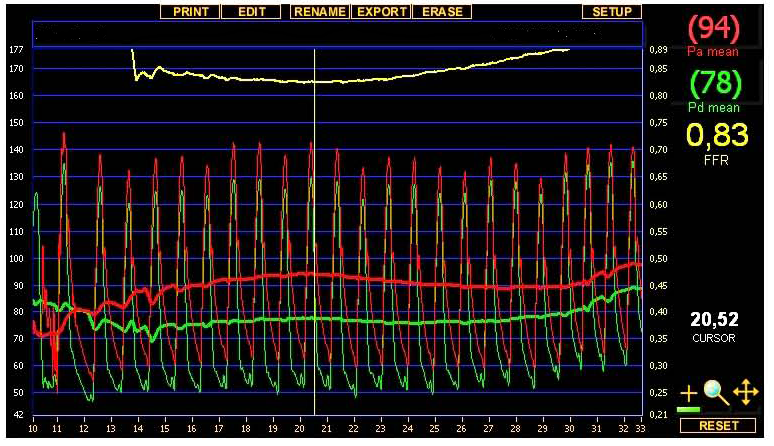
**Pressure wire measurement of the left anterior descending artery: during the ic. injection of 100 μg of adenosine, only a nonsignificant translesional pressure gradient was provoked**. The ratio of the pressure (FFR: fractional flow reserve) detected distally to the stenosis (green line) and the proximal (aortic) pressure (red line) was 0.83.

A 62-year-old male, who had undergone bilateral mammary bypass grafting on the LAD and the right coronary artery (RCA) 4 years earlier. Coronary angiography showed an almost occluded LIMA graft (string sign) (Figure 2A) with 50-60% stenosis on the proximal part of the LAD (Figure 2B). The anatomosis of the right IMA graft displayed a high-grade stenosis. The previous myocardial scintigraphy revealed a reversible perfusion defect on the inferior wall, while pressure wire measurement on the LAD proved that the lesion of the LAD was not associated with a significant pressure gradient even during vasodilatation (FFR = 0.83) (Figure 3), suggesting sufficient remaining (competitive) flow in the native LAD. A stent was implanted in the right IMA-RCA anastomosis, after which the patient's symptoms improved.

### Case 3

**Figure 4:**
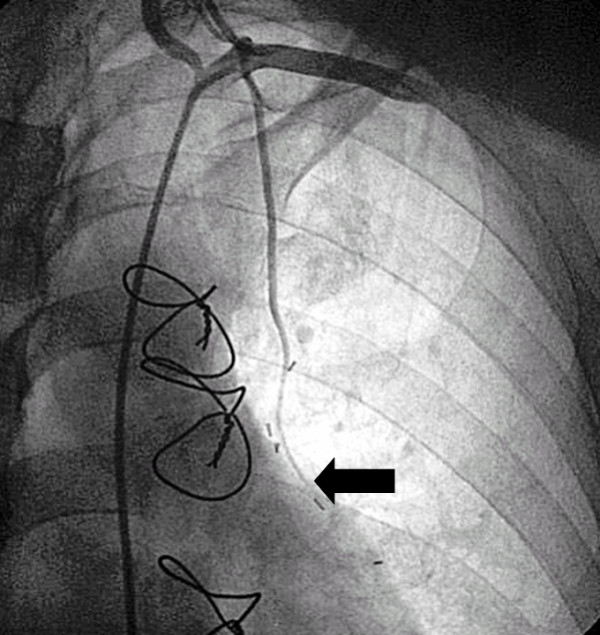
**String sign of the left internal mammary artery graft (black arrow)**.

**Figure 5:**
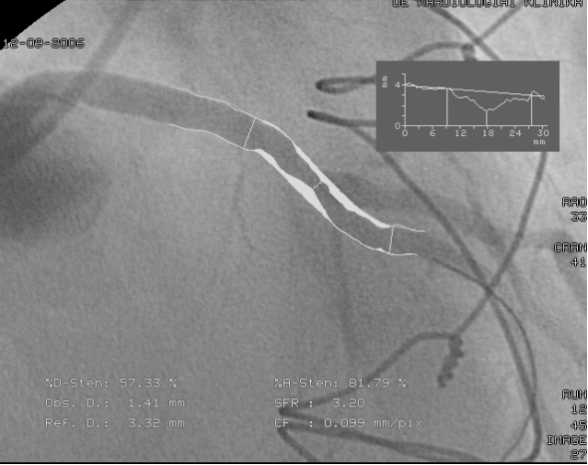
**Quantitative coronary analysis measurement of the left anterior descending artery lesion demonstrated 57% diameter stenosis on the left anterior descending artery**. However, the pressure wire sensor (black arrow) detected only a nonsignificant translesional gradient during vasodilatation (FFR = 0.89), indicating appropriate competitive flow in the LAD.

A 61-year-old man after an inferior myocardial infarction underwent surgical revascularization involving a LIMA graft on the LAD and a venous graft on the aberrant left circumflex artery (LCx) from the right Valsalva sinus. One year later, the repeated coronary angiography detected string sign of the LIMA graft (Figure 4.) and total occlusion of the venous graft. Despite the 57% diameter stenosis of the LAD lesion (Figure 5.), pressure wire measurement excluded a hemodynamically significant lesion in this coronary artery. The aberrant LCx lesion was treated percutaneously by stent implantation.

### Case 4

**Figure 6:**
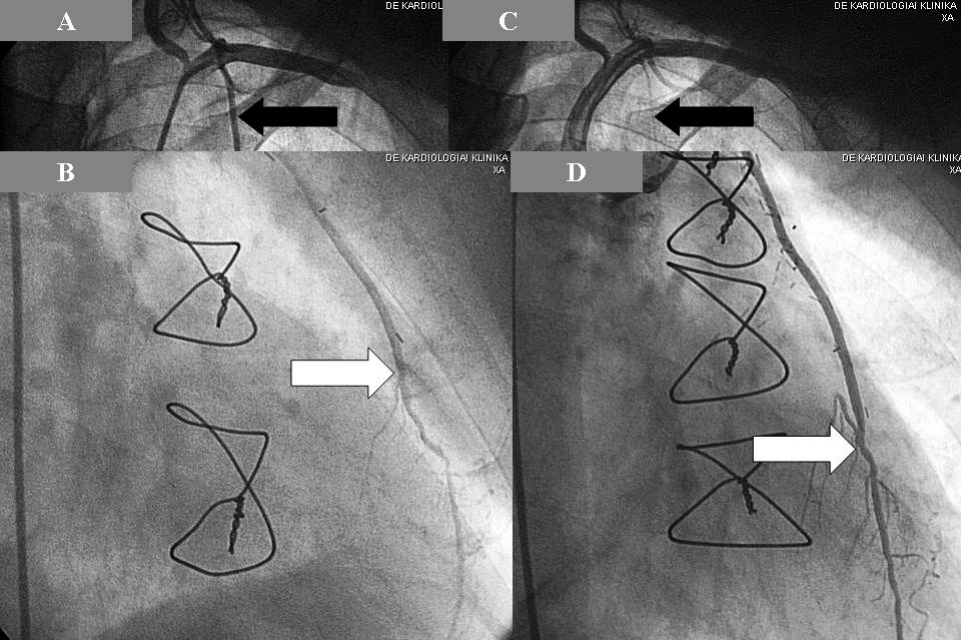
**Mammary graft before and after the reoperation with the radial graft: **A,B**: The left internal mammary artery graft was widely open before the reoperation (black arrow: left internal mammary artery, white arrow: anastomosis of the left internal mammary artery graft to the left anterior descending artey)**. **C**: After the reoperation involving a radial graft, the string sign developed on the previously patent left internal mammary artery graft (black arrow). **D**: The anastomosis of the radial graft is indicated by the white arrow. This radial anastomosis was applied more distally than the anastomosis of the mammary graft. The new bypass was administered on the left anterior descending artery as there was considered to be a significant lesion under the left internal mammary artery graft.

Five years after the bypass operation (LIMA-LAD, SVGA-1^st ^obtuse marginal branch (OM), -RCA), a 65-year-male patient was recatheterized because of the recurrence of angina-like symptoms. The venous grafts were occluded, while the LIMA graft was widely patent, with an intermediate lesion in the run-off under the anastomosis of the LIMA. A total arterial revascularization redo operation was decided on, and radial grafts were applied on the LAD, OM and RCA. After 3 years repeated coronary angiography revealed the string sign of the previously patent LIMA graft because the competitive flow of the new radial graft supplied the LAD more intensively than the previous LIMA graft (Figure 6).

### Case 5

**Figure 7:**
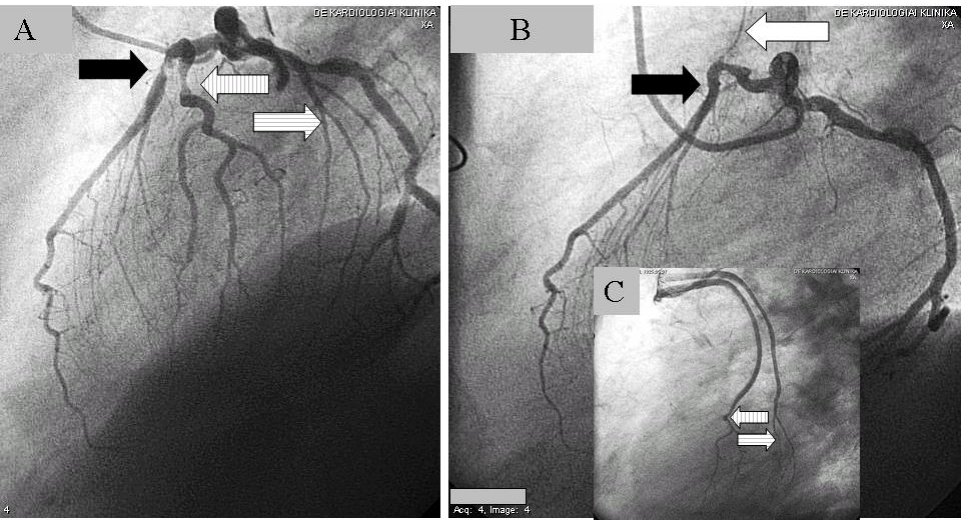
**String phenomenon of the internal mammary artery graft, caused by competitive flow of the left anterior descending artery because of regression of the left anterior descending artery lesion**. **A**: Severe stenosis (as a consequence of non-occlusive thrombus formation associated with a ruptured plaque) on the left anterior descending artery (black arrow) just under the origin of a well-developed diagonal branch (left striped arrow), and on an obtus marginal of the left circumflex artery (right striped arrow). **B**: Three years after the bypass surgery, the contrast injection of the left coronary artery shows retrograde filling of the degenerated left internal mammary artery graft (white arrow). There is no significant stenosis at the site of the original lesion (black arrow), presumably because of the healing of the ruptured plaque with resolution of the thrombus. After the bypass surgery, the grafted diagonal and marginal branches became occluded, but the applied venous grafts provided good filling of the run-off (**C**: striped arrows).

Three years after the bypass surgery (LIMA-LAD, SVGA-1^st ^diagonal, -1^st ^OM and -RCA), a 75-year-old man underwent recatheterization because of angina-like symptoms. Coronary angiography showed the string sign of the LIMA graft, while the venous grafts were patent (Figure 7). The originally severe lesion demonstrated such a degree of regression that the flow in the LAD improved to a normal level, resulting in competition of the LIMA graft.

### Case 6

**Figure 8:**
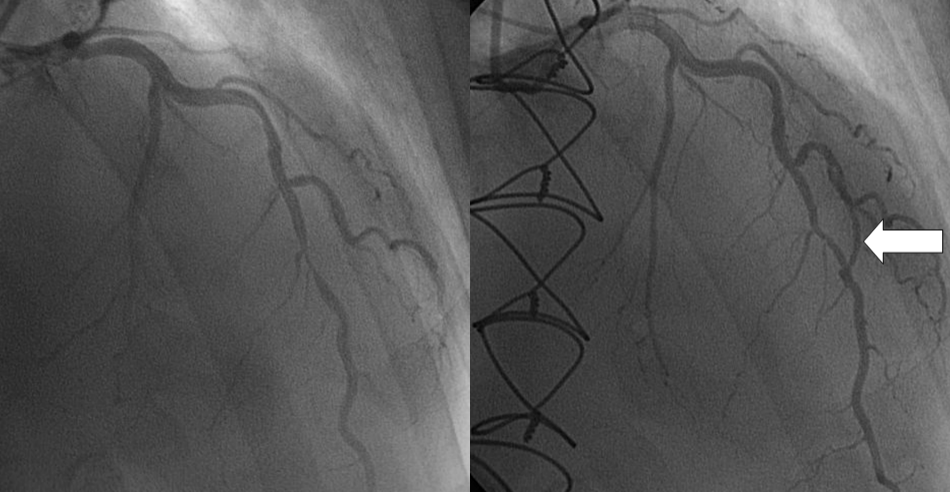
**The left side shows the tight lesion of the middle left anterior descending artery before the coronary artery bypass grafting, while the right panel reveals the situation 3 years after the operation, with the short open distal part of the left internal mammary artery graft (arrow) bypassing the proximal part of the left anterior descending artery to the distal segment through the diagonal branch**. The proximal part of the left internal mammary artery graft has been occluded because of the competitive flow through this connection. A slight regression of the originally very severe lesion may also be observed.

A 56-year-old male patient had presented with unstable angina 3 years previously. Angiography revealed three-vessel disease with occlusion of the RCA and the LCx. In the middle region of the LAD, there was a tight lesion, and the orifice of the diagonal branch exhibited a mild lesion. The patient underwent bypass grafting: the LCx and the RCA received radial artery grafts, while the diagonal branch and the LAD were sequentially grafted by the LIMA. The patient made a good recovery from surgery, and was free of symptoms for more than 2 years. The index admission of the patient was necessary because of recurrence of the angina. The repeated angiography demonstrated patent radial grafts to the RCA and the LCx, but the LIMA graft was occluded proximally and did not fill the diagonal branch. Only the short distal part of the LIMA graft was open, connecting the proximal part of the LAD to the distal segment through the diagonal branch (Figure 8). It was decided to perform an intervention on the LAD lesion with stent implantation in order to improve the flow in the distal LAD.

## Conclusion

Internal mammary arteries are the grafts of choice for CABG because they are generally free of atherosclerosis and exhibit high patency rates as grafts. Despite the overall excellent patency rates, however diffuse or distal narrowing of IMA grafts, the string phenomenon, occurs with appreciable frequency, and ultimately leads to graft failure [5-8].

It has been shown that CABG to minimally and moderately stenotic target vessels may lead to competitive flow between the native vessel and the graft. Competitive flow is a multifactorial phenomenon, and even an estimate of its impact is difficult, especially if based on preoperative coronary angiography alone [9,10].

There is a significant body of clinical evidence suggesting that the size of the IMA is reduced under competitive flow conditions, and this can result in IMA graft failure. The study by Pagni et al. implicated two factors that could potentially lead to the development of IMA graft failure: retrograde systolic flow and low diastolic flow. Endothelial signals may modulate downsizing of the vessel diameter when faced with either or these stimuli [14].

Our series of patients was found to include 6 cases with the string sign of the LIMA. Angiography indicated competitive flow in all 6 cases as a cause of the development of the failure of the LIMA graft. In the cases without redo graft on the LAD, QCA measurement revealed 31-57% diameter stenosis in the LAD proximally to the LIMA graft. In 2 patients, we performed FFR measurements to confirm the nonsignificance of the LAD lesions. The FFR values (0.83 and 0.89) excluded s significant flow limitation of the stenoses. Of the 6 cases demonstrating the string sign, 3 (50%) had nonsignificant stenosis according to the FFR or QCA criteria.

The FFR (calculated from coronary artery pressure measurements) has been shown to be an accurate and lesion-specific index for determining if an intermediate stenosis is functionally significant and responsible for reversible ischemia. A threshold value of 0.75 clearly distinguishes lesions responsible (FFR < 0.75) or not responsible (FFR ≥ 0.75) for reversible ischemia during exercise. On the basis of the published data, our observations are in line with the value of the functional assessment of lesion severity by FFR measurement as a potential guide for a decision concerning grafting of an intermediate lesion [15].

In 3 cases, we identified relatively rare sources of the competitive flow in the background of the string sign: a new radial graft on the LAD, significant regression of the LAD lesion, and a sequential LIMA graft to the diagonal branch without a significant lesion.

Our results suggest that the most common cause of the string sign is a functionally nonsignificant lesion on the target vessel, but other scenarios leading to competitive flow must also be considered. Table 1 summarizes the causes of the string-sign formation and the applied investigations for confirmation of the competitive flow.

**Table 1:** The summery of the causes of the string-sign and the applied investigations for confirmation of the competitive flow

Cases	CABG	QCA stenosis % in the LAD proximally to the LIMA anastomosis	Cause of the competitive flow	Proof of the competitive flow
**1**	LIMA-LAD SVGA-RCA	48%	Non- significant LAD lesion + patent LIMA side branch	angiogram

**2**	LIMA-LAD RIMA-RCA	53%	Non-significant LAD lesion	angiogram + FFR = 0.83

**3**	LIMA-LAD SVGA-LCx	57%	Non-significant LAD lesion	angiogram + FFR = 0.89

**4**	1st: LIMA-LAD, SVGA-1st OM, SVGA-RCA 2nd: rad-LAD, rad-LAD, SVGA-RCA	100%	The concomitant radial graft to the LAD	angiogram

**5**	LIMA-LAD, SVGA-1^st ^diag., SVGA-1^st ^OM, SVGA-RCA	31% (87% before the operation)	Regression of the LAD lesion	angiogram

**6**	LIMA-diag-LAD, rad-LCx, rad-RCA	62% (83% before the operation)	Non-significant lesion on the diagonal branch	angiogram

Even though previous observations did indicate clinical relevance of an occluded bypass graft on a nonsignificant lesion, we consider that careful analysis of the clinical scenarios is mandatory for a correct decision regarding grafting: an operation that is too early may lead to the inappropriate use of grafts without prevention of the consequences of the future progression of the disease.

## Consent

Written informed consent was obtained from patients.

## Competing interests

The authors declare that they have no competing interests.

## Authors' contributions

RK participated in the design and coordination of the study. ZG participated in coordination of the study. TU participated in evaluating the results. GS participated in choosing the cases. IR participated in choosing the cases. TS participated in choosing the cases. IH participated in drawing conclusions. IE participated in coordination of the study. AP participated in choosing the cases. ZK participated in all part of the study. All authors read and approved the final manuscript.
